# The Influence of the COVID-19 Pandemic Emergency on Alcohol Use: A Focus on a Cohort of Sicilian Workers

**DOI:** 10.3390/ijerph20054613

**Published:** 2023-03-05

**Authors:** Emanuele Cannizzaro, Luigi Cirrincione, Ginevra Malta, Santo Fruscione, Nicola Mucci, Francesco Martines, Fulvio Plescia

**Affiliations:** 1 Department of Health Promotion Sciences, Maternal and Child Care, Internal Medicine and Medical Specialties ‘Giuseppe D’Alessandro’, University of Palermo, Via del Vespro 133, 90127 Palermo, Italy; 2 Department of Experimental and Clinical Medicine, University of Florence, Largo Brambilla, 3, 50134 Florence, Italy; 3 Department of Biomedicine, Neuroscience and Advanced Diagnostics (BiND), Section of Audiology, University of Palermo, Via del Vespro 129, 90127 Palermo, Italy

**Keywords:** alcohol, stress, workers, AUDIT-C, COVID-19

## Abstract

The period between the beginning and the end of the COVID-19 pandemic emergency generated a general state of stress, affecting both the mental state and physical well-being of the general population. Stress is the body’s reaction to events or stimuli perceived as potentially harmful or distressing. Particularly when prolonged over time, it can promote the consumption of different psychotropic substances such as alcohol, and thus the genesis of various pathologies. Therefore, our research aimed to evaluate the differences in alcohol consumption in a cohort of 640 video workers who carried out activities in smart working, subjects particularly exposed to stressful situations due to the stringent rules of protection and prevention implemented during the pandemic. Furthermore, based on the results obtained from the administration of the AUDIT-C, we wanted to analyse the different modes of alcohol consumption (low, moderate, high, severe) to understand whether there is a difference in the amount of alcohol consumed that could predispose individuals to health problems. To this end, we administered the AUDIT-C questionnaire in two periods (T_0_ and T_1_), coinciding with annual occupational health specialist visits. The results of the present research showed an increase in the number of subjects consuming alcohol (*p* = 0.0005) and in their AUDIT-C scores (*p* < 0.0001) over the period considered. A significant decrease in subgroups who drink in a low-risk (*p* = 0.0049) mode and an increase in those with high (*p* = 0.00012) and severe risk (*p* = 0.0002) were also detected. In addition, comparing the male and female populations, it emerged that males have drinking patterns that lead to a higher (*p* = 0.0067) health risk of experiencing alcohol-related diseases than female drinking patterns. Although this study provides further evidence of the negative impact of the stress generated by the pandemic emergency on alcohol consumption, the influence of many other factors cannot be ruled out. Further research is needed to better understand the relationship between the pandemic and alcohol consumption, including the underlying factors and mechanisms driving changes in drinking behaviour, as well as potential interventions and support strategies to address alcohol-related harm during and after the pandemic.

## 1. Introduction

11 March 2020 has now become a historic date. On that day, the World Health Organisation (WHO), following a careful analysis of the risks associated with the spread of severe acute respiratory syndrome by a coronavirus (SARS-CoV-2), declared that the COVID-19 epidemic could be considered a real pandemic [1,2,3,4]. Since then, the world’s population has had to change its lifestyle, aligning with the rules laid down by the various governments [e.g., Italian, British, French, American] [5,6,7,8] concerning the prevention and protection methods to be implemented in private life, in public places, in school and university environments and in the workplace [9,10,11,12,13,14,15]. Moreover, this health emergency has forced public and private administrations to resort to smart-working, or agile working, as a suitable method to manage and contain the pandemic. 

These changes have had a profound impact on working and social life. All this, together with the continuous evolution of rules to be followed, has led to the genesis of a condition of general consistent malaise that has facilitated the beginning of various forms of stress and related disorders [16,17,18,19,20,21,22], including the one now identified as ‘COVID-19 stress’ [23].

Stress is a generic term often used to indicate adverse life conditions [24]. Exposure to a stressful stimulus over a long period can promote the onset of different moods such as anxiety, fear, anger, excitement, and sadness that can, in the case that they exceed the individual’s coping abilities, promote the occurrence of different pathologies [25,26,27,28,29] and increase vulnerability to use of substances of abuse [30,31,32].

Furthermore, continued exposure to aversive stimuli is influenced by different contexts, such as education (school, university) and work [33,34,35,36]. It has been pointed out that the working environment, its organisation, and work-related behaviour are themselves stressors, and as such can influence workers’ psychological well-being [37]. Recently, different research has focused on the relationship between stress at work, aggravated by the new prevention and protection guidelines due to the pandemic emergency, and the development of mental disorders and risk behaviours such as the use of substances of abuse [37,38]. In this context, the risk of developing such conditions is related to the type of work performed, the potential for social interaction (prolonged or not), and exposure to different environmental contaminants that would promote the genesis of other pathologies. 

Notably, among the addictive behaviours related to stressful conditions, alcohol abuse leads the way due to alcohol’s easy obtainability and organoleptic properties [39,40]. In this context, additional scientific evidence shows that people who experience periods of severe economic or psychological stress are more inclined to consume alcoholic beverages with the consequent onset of abuse and addiction behaviour [41,42]. The pandemic has led to changes in alcohol consumption patterns, with some individuals drinking more due to increased stress and isolation. In contrast, others have reduced or abstained due to health or financial concerns. Interesting research by Sohi and colleagues has shown that during the pandemic, the amount and mode of alcohol intake are substantially heterogeneous and depend on the country in which the research was conducted. These authors suggest that further research is needed to understand better the relationship between the pandemic and alcohol consumption, including the underlying factors and mechanisms driving changes in drinking behaviour, and to create potential interventions and support strategies to address alcohol-related harm during and after the pandemic [43]. 

Based on the aforementioned, this research aimed to assess how both the approach and the mode of consumption of alcoholic beverages changed during the pandemic period in a population of video workers who were forced by the pandemic to carry out activities in smart working. Before administering the AUDIT-C questionnaires, we excluded part of the population based on different criteria. In particular, we decided to exclude subjects with a body mass index above or equal to or greater than 32, with dysmetabolic and oncological pathologies. This decision stems from knowledge of these variables’ influence on alcohol consumption. On the other hand, it has been reported that individuals with a high BMI, particularly those with obesity, are at increased risk of developing dysmetabolic pathologies such as diabetes, metabolic syndrome, and non-alcoholic fatty liver disease, which in turn can increase the risk of developing alcohol addiction by altering the body’s response to alcohol and affecting brain’s reward pathways [44,45]. Moreover, some cancer treatments such as chemotherapy can be less effective in individuals who consume alcohol. Therefore, it is probable that individuals with an oncological pathology will limit or avoid alcohol consumption to reduce the risk of cancer progression and other health complications [46]. 

Finally, based on the data collected through the administration of the AUDIT-C test, it was possible to classify the population into different categories that accounted for the risk of encountering pathologies related to improper consumption of alcohol. 

Given the scientific evidence on the increasing consumption of alcoholic beverages, the hypothesis of our study focuses on the idea that the pandemic period, marked by stringent norms of prevention and protection, was a risk factor that could exert such pressure as to influence the mode of alcohol consumption as much as the amount of alcohol consumed.

## 2. Materials and Methods

### 2.1. Experimental Design

This observational study was conducted on a cohort of video workers, considering the recommendations indicated by the Strengthening the Reporting of Observational Studies in Epidemiology (STROBE) [47]. The sample of this study is “opportunistic” because data were collected based on the availability of participants at a private practice of occupational medicine in Palermo, Italy. 

The study was conducted in two different periods: T_0_: June 2020 and T_1_: April 2022, the date on which, given the end of the emergency state (31 March 2022), apart from vulnerable subjects, the majority of the working population was considered to have returned to work in person. 

For this study, subjects of both sexes aged between 25 and 65 years with a video work history of at least four years were enrolled. The population was initially 800 (T_0_) workers (400 males and 400 females). Among these, 11% (63 F and 25 M) refused to participate in the study, and 6% (11 F and 37 M) were excluded from the study because they did not show up for the specialist visit as they were no longer employed by their companies or were absent due to illness or other causes. A further 3% (9 F and 15 M) of subjects who, at the time of the first medical examination, were on drug therapy for anxiety disorders, depression, or other psychiatric disorders, were also excluded; workers with a body mass index greater than or equal to 32, employees on drug therapy for dysmetabolic pathologies, all workers with previous or current oncological pathologies, and workers with a previous history of pathological addictions were also excluded. All subjects admitted to the study met the inclusion criteria considered in our study. 

Eventually, the study enrolled 640 adults: 321 males and 319 females (M/F ratio 1.006). The number of subjects analysed at time T_0_ was identical to that of T_1_, although their number varied within the subgroups considered in this study. 

At the end of the patients’ general anamnesis, all participants were asked to fill in a questionnaire to establish their alcohol consumption patterns and degree of alcohol dependence.

All participants were informed about the purpose of the study and signed the informed consent before participating. Respondents were asked not to mention their or the organisation’s names in the questionnaire to ensure privacy and anonymity. 

All data have been handled according to Italian law to protect privacy (Decree No. 196, January 2003). A multidisciplinary team of health experts collected and analysed the data through the questionnaires administered on alcohol habits.

### 2.2. Assessment of Alcohol Consumption and Degree of Dependence

The assessment of alcohol consumption and the relative risk associated with its use was conducted by administering the Alcohol Use Disorders Identification Test-Concise (AUDIT-C), a modified version of the 10-question Alcohol Use Disorders Identification Test (AUDIT) developed by the World Health Organisation. This test is valuable for investigating alcohol consumption and how it occurs. It also allows us to identify patients who are hazardous drinkers and those who are particularly at risk of developing alcohol-related disorders. 

This instrument is a 3-item survey with a total score ranging from 0 to 12 points. Each item has five response options ranging from 0 to 4 points. A score of 3 or more points on the AUDIT-C may indicate that people are risk drinkers or have alcohol use disorders. A score of 4 or more for men and 3 for women is predictive of potential alcohol abuse. A person’s likelihood of developing an alcohol use disorder is directly proportional to a higher test score [48]. 

Furthermore, based on the score obtained from the AUDIT-C test, we divided our population into five different categories: abstainer (score = 0), low risk (score = 1–3 men; 1–2 female), moderate risk (score = 4 men; 3–4 female); high risk (score = 5–7 men and female) and severe risk (8–12). The groups were structured based on previous research on the association between alcohol intake and health risks [49,50,51,52]. 

### 2.3. Statistical Analysis

The statistical analysis of the data was conducted using the GraphPadPrism 8.01 statistical software package (GraphPad Company, San Diego, CA, USA). Initially, the collected data were analysed to understand whether they were normally distributed and, consequently, to choose the most suitable statistical analysis to apply. To do this, we applied the D’Agostino–Pearson omnibus normality. Given that our data did not follow a normal distribution, we used the non-parametric Chi-square test to determine whether the frequency values obtained with the survey were significantly different from those obtained with the theoretical distribution. Specifically, the Chi-square test was applied to understand whether there were differences in the number of total consumers and between the male and female samples in the two periods, and to assess possible variations in the risk categories obtained from the analysis of the AUDIT-C test data over the time interval considered. Moreover, logistic regression was also performed to calculate the probability of the association between alcohol consumption and gender. Data are expressed as odds ratio (OR).

The Wilcoxon test was applied for paired data, and the Mann–Whitney U test was used for unpaired data to assess the differences in AUDIT-C scores among the population under our study. A descriptive analysis of the data obtained was also conducted to understand the consumption pattern and the amount of alcohol consumption. Data were reported as mean with 95% CI. Statistical significance was set at *p* < 0.05.

## 3. Results

### 3.1. Alcohol Consumption in the General Population

The collection and analysis of data useful for the identification of the number of subjects consuming alcohol and their risk of developing problems related to the misuse of the substance were conducted by the administration of the AUDIT-C. 

In detail, within the sample analysed, a more significant number of subjects who consumed alcoholic beverages, both at T_0_, 467 (72.97%; audit score (AS) 3.229, confidence interval (CI) 3.072–3.386) and at T_1_, 519 (81.09%; AS 3.925, CI 3.746–4.104) compared to those who claim not to drink at both T_0_, 173 (27.97%) and T_1_, 121 (18.91%) was highlighted. Moreover, among the subjects consuming alcoholic beverages, there were differences regarding the percentage of subjects who consume alcohol with different risk modes. Indeed, the subjects who consume alcohol in a manner considered to be a low risk both at T_0_, 298 (63.81%; AS 2.168, CI 2.069–2.258) and at T_1_, 245 (47.21%; AS 2.139, CI 2.032–2.245) prevail over those who consume it in a riskier manner (Table 1). 

**Figure 1:**
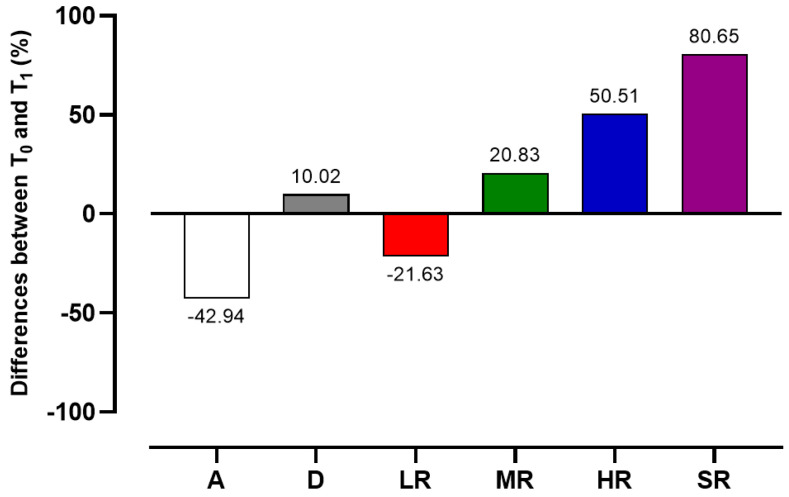
Differences in the percentage of subjects who consumed alcohol in the two times considered. A = Abstainers; D = Drinkers; LR = Low Risk; MR = Moderate Risk; HR = High Risk; SR = Severe Risk.

When we analysed the consumption of alcoholic beverages in a subgroup of drinkers, the descriptive analysis of the data showed that there was a reduction in the percentage of the number of low-risk subjects and an increase in those at moderate, high and severe risk between the time intervals analysed (Figure 1). 

**Figure 2:**
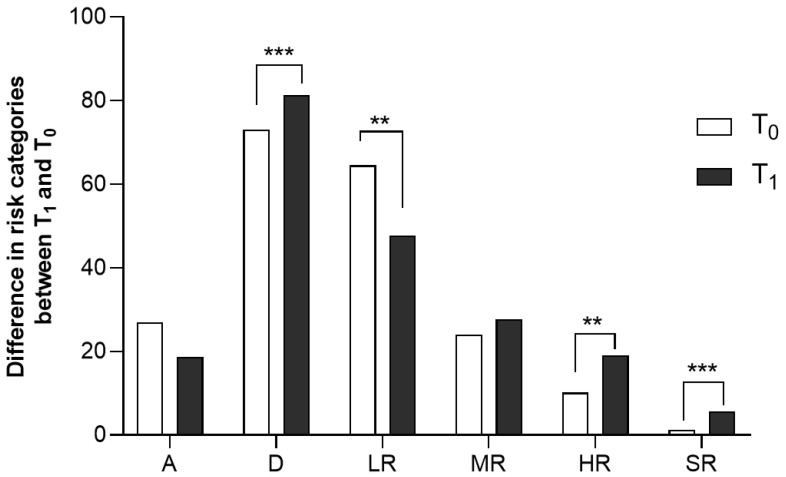
Differences in risk percentages over the time interval considered in our study, T_1_ vs. T_0_. A = Abstainers; D = Drinkers; LR = Low Risk; MR = Moderate Risk; HR = High Risk; SR = Severe Risk. ** *p* < 0.01; *** *p* < 0.001 vs. T_0_.

Considering the data obtained from the descriptive analysis, we assessed whether there were differences in the number of consumers and those belonging to the different risk categories in the two periods considered. In detail, statistical analysis by the Chi-square test showed a significant increase in the percentage of total consumers (χ^2^ = 11.94, z = 3.455, *p* = 0.0005). The analysis of the data on the number of subjects consuming alcohol in different risk modes revealed a reduction in the percentage of subjects consuming alcohol in a low-risk manner (χ^2^ = 7.915, z = 2.813, *p* = 0.0049) and an increase in the high (χ^2^ = 10.54, z = 3.247, *p* = 0.0012) and severe (χ^2^ = 13.92, z = 3.731, *p* = 0.0002) risk groups at T_1_ compared to T_0_. There were no significant differences in the percentage of moderate-risk drinkers between T_1_ and T_0_ (χ^2^ = 0.8292, z = 0.9106, *p* = 0.3625) (Figure 2).

### 3.2. Differences in Alcohol Consumption between Males and Females

Given the data obtained on drinking behaviour in the sample analysed, we wondered whether there were differences between the percentages of male and female subjects regarding alcohol consumption and differences in the risk related to alcohol consumption (Table 2). 

The analysis conducted by applying the Chi-square test did not reveal any significant differences in the percentages of alcohol drinkers between males and females (χ^2^ = 1.230, z = 1.109, *p* = 0.2675; χ^2^ = 1.150, z = 1.072, *p* = 0.2836) in the two timeframes considered. 

**Figure 3:**
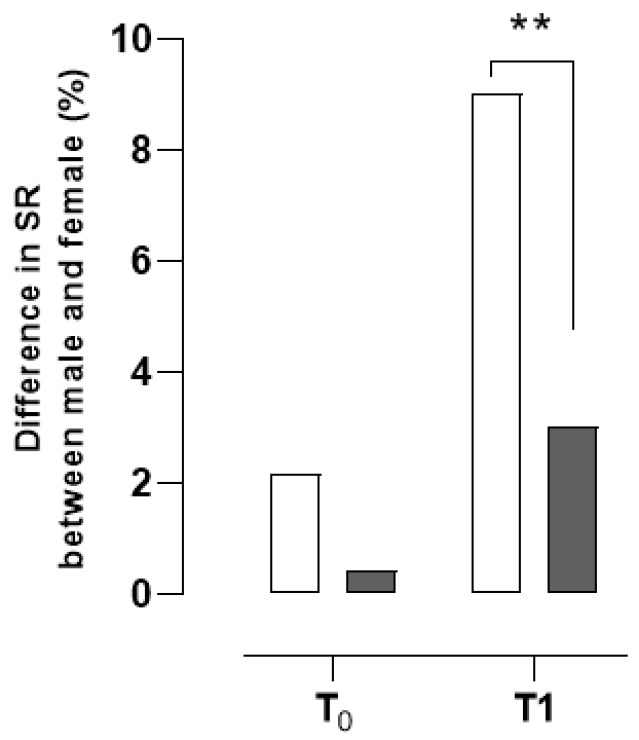
Differences in severe risk percentages between males and females. T_0_, T_1_ = times of data collection and analysis. ■ = Female; ☐ = Male. ** *p* < 0.01; vs. Female.

When we evaluated the differences in the consumption of alcoholic beverages obtained from the analysis of the AUDIT-C test, the analysis of the data by the Chi-square did not reveal statistically significant differences both at T_0_ and at T_1_ between males and females regarding low risk (χ^2^ = 0.05162, z = 0.2272, *p* = 0.8203; χ^2^ = 0.7793, z = 0.8828, *p* = 0.3774), moderate (χ^2^ = 0.3778, z = 0.6146, *p* = 0.5388; χ^2^ = 0.06674, z = 0.2583, *p* = 0.7961) and high (χ^2^ = 0.06298, z = 0.2509, *p* = 0.8019; χ^2^ = 0.1887, z = 0.4344, *p* = 0.6640). When we went to analyse the data concerning the drinking mode, the data analysis showed that at T_0_, there were no differences (χ^2^ = 2.823, z = 1.680, *p* = 0.0929) between males and females. On the contrary, at time T_1_, we found statistically significant differences in the percentage of males drinking in a manner that exposes them to a severe health risk (χ^2^ = 7.350, z = 2.711, *p* = 0.0067) compared to females (Figure 3). 

Furthermore, based on the data obtained, we calculated the probability of the association between alcohol consumption and gender in the two time periods considered. Specifically, there was no more of a significant probability of drinking in male subjects than in female subjects in the two times considered (OR: 0.9863—95% CI: 0.9148–1.063; OR: 1.042—95% CI: 0.9763–1.112).

We also analyzed the differences in the AUDIT-C score between T_0_ and T_1_. Statistical analysis was conducted using the Wilcoxon test to understand any differences in the AUDIT-C score in the two times covered by our study. The analysis showed a significant increase in the score at time T_1_ (*p* < 0.0001) compared to that obtained at time T_0_.

## 4. Discussion

The pandemic emergency experienced in recent years has drastically changed many aspects of daily life. The two waves of the contagion, which occurred over a relatively short period of time, have led to isolation, forced living in confined spaces and profound changes in everyone’s working life [15,53]. All of these things have exerted intense pressure on the adaptive capacities of the population; while in the first phase, these capacities served to cope with adversity by drawing on our instinctive spirit of survival, with the prolongation of the pandemic, they have fostered the development of a condition of persistent stress which may alter an organism’s internal homeostasis and lead to the onset of different pathologies and/or the establishment of addictive behaviour. This may include an increase in alcohol consumption [21,54,55]. 

The trend recorded for the consumption of alcoholic beverages is well in line with the data obtained from the present observational study, in which there was an increase in the number of alcoholic drinkers (+10.02%) over the time interval examined. It is also interesting to note that the percentage of subjects who consume alcohol is always higher (72.97%; 81.09) than those who claim not to drink (27.03%; 18.91%). 

The result concerning alcohol consumption behaviour is a sobering thought. In particular, the data showed a different pattern of alcohol consumption. Specifically, following analysis of the subgroup categories, a reduction in the number of subjects who consume alcohol in a manner that exposes them to a low health risk emerged both in males (−32.14%) and females (−12.78%) over the time interval considered. In addition, a significant increase in the number of subjects who consume alcohol in a manner that exposes them to higher (52.51%) and severe (80.65%) health risks were revealed.

In addition to the increase in the consumption of alcoholic beverages, our data also showed an increase in the AUDIT-C score, both when we evaluated all the population subjects of our study (*p* < 0.0001) and when we analysed the subgroup of drinkers (*p* < 00001). An increase in the AUDIT-C score can predict the development of physical or social problems related to alcohol consumption. In particular, there are risks associated with alcohol consumption that may vary depending on gender, age, general health and the amount and frequency of alcohol consumption. Excessive alcohol consumption can have serious adverse health consequences and increase the risk of liver disease, pancreatitis and certain types of cancer. It can also lead to different mental health problems such as depression and anxiety [56].

Increased consumption of alcoholic beverages and changes in the mode of consumption can be traced back to the emotional distress experienced during the COVID-19 pandemic. During this period, a large part of the population had to drastically change their daily routines, starting with their mode of work. 

Remote working has, for example, encouraged social isolation and the onset of general malaise due to the impossibility of setting up a good workplace and/or reconciling work and private commitments effectively [57,58,59]. 

The office is a space, but it is, above all, a community. Working in the office means being surrounded by workers, collaborating, asking for help, chatting over a coffee, and having pure and simple human contact that makes us feel part of a group. Working from home means giving up completely the social and human component of office work.

Working from home and limiting opportunities for sociability and collaboration can only lead to a growing sense of isolation and increased health risks. This analysis may seem overly alarmist and pessimistic, but it is confirmed by various studies [59,60,61,62,63]. It has been shown that homeworking aligns with workers’ satisfaction only if it is not protracted for a long time. In fact, after an initial period of enthusiasm, there is a widespread desire to return to office life, even in the face of losing time and money for travel [64]. The reason for this choice is mainly the feeling of loneliness that affects home workers [65].

In-person working also underwent profound changes due to the implementation of multiple measures to contain the contagion [66,67]. All of the above were able to generate a solid stimulus to interrupt the normal internal balance of the body and make the condition experienced highly stressful. 

This condition may partly explain the increase in the consumption of alcoholic beverages in a manner that exposes health risks, as was recorded in our study.

Stress is a factor closely correlated with often uncontrolled consumption of alcohol and with relapses back into its use after a period of abstinence [68]. Different studies have shown that particularly dangerous and demanding work environments and family stress are factors associated with increased alcohol consumption [69,70,71,72]. This is partly attributable to increased cortisol release which is triggered by activation of the hypothalamic-pituitary–adrenal axis, one of the main modulators of the adaptive stress response [73]. In particular, impaired regulation of the HPA axis is associated with problematic alcohol consumption, and the nature of this dysregulation varies with the stages of progression towards alcohol dependence [74,75,76]. 

The motivation that drives people to consume more and more alcohol can be traced back to the molecule’s action. In fact, alcohol exerts anxiolytic effects, and its intake promotes a reduction in the perception of stress [77,78]. Alcohol can modulate the activation of the hypothalamic–pituitary–adrenal (HPA) axis both directly and indirectly, resulting in a different regulation of glucocorticoid release and the consequent alteration of the adaptive stress response [79,80,81]. 

This reduction in the state of tension facilitated by alcohol intake is attributable to its ability to stimulate the action of different inhibitory neurotransmitters, such as γ-aminobutyric acid [GABA] and opioids. These, through inhibition of the hypothalamus’s paraventricular nucleus (PVN), modulate the release of neuropeptides that are helpful in stimulating the synthesis and subsequent release of cortisol [72,82,83,84], thereby attenuating the stress response. 

Alcohol can thus assume a positive reputation among the general population, who may use it as ‘self-medication’ to combat incredibly unpleasant living conditions and sources of stress. This encourages a growing amount of alcohol to be consumed, thereby promoting an increased risk of alcohol-related diseases.

## 5. Limitations of the Study

Although this research provides further evidence of the influence of stress on alcoholic beverage consumption, it does not lack some limitations that could be considered for future studies. In particular, the study, although carried out on a reasonably homogeneous population, could not consider the correlation between the stress biomarkers assessed at the times considered and alcohol consumption patterns. This would have provided intriguing evidence of the risk of alcohol-related disease in the population examined. 

## 6. Conclusions

Our study highlighted the way in which the imposition of smart working during the pandemic was one of the factors that negatively impacted the psycho-physical wellbeing of workers by causing stress that encourages the onset of risky behaviour. 

In the population examined, it emerged that during the COVID-19 pandemic, the number of alcohol users and the modes of consumption of alcoholic beverages changed. From our study, the increase in alcohol consumption in ways that increase health risk is a result to be treated with particular concern, and to which we should pay particular attention. 

This result was related to difficult working conditions, which are a source of intense stress. In-depth knowledge of the risky ways in which an individual worker consumes alcohol can enable the implementation of preventative actions to safeguard their health and to improve the safety of the worker and those who work with them.

Further studies are necessary to determine the close correlations between work-related stress and risky alcohol consumption in individual video workers, especially after the COVID-19 pandemic.

## Figures and Tables

**Table 1:** Data on the use of alcoholic beverages and the risk of alcohol-related problems. T_0_, T_1_ = times during which the tests were administered and completed; n° = number; % = percentage; AUDIT-C = Alcohol Use Disorders Identification Test-Concise; CI = Confidence interval.

Sample				
T_0_	n°	%	AUDIT-C Score Index Mean	95% CI
	640	100	2.199	2.199–2.520
				
Abstainers	173	27.03		
Drinkers	467	72.97	3.229	3.072–3.386
				
Low Risk	298	63.81	2.168	2.069–2.258
Moderate Risk	114	24.41	4.386	4.295–4.477
High Risk	49	10.49	6.592	6.449–6.734
Severe Risk	6	1.28	8.167	7.738–8.595
				
**T_1_**	**n°**	**%**	**AUDIT-C Score Index Mean**	**95% CI**
	640	100	3.152	2.966–3.337
				
Abstainers	121	18.91		
Drinkers	519	81.09	3.925	3.746–4.104
				
Low Risk	245	47.21	2.139	2.032–2.245
Moderate Risk	144	27.75	4.340	4.241–4.392
High Risk	99	19.08	6.384	6.286–6.481
Severe Risk	31	5.97	8.258	8.090–8.407

**Table 2:** Data on the use of alcoholic beverages and the risk of alcohol-related problems differentiated between males and females. T_0_, T_1_ = times during which the tests were administered and completed; n° = number; % = percentage; AUDIT-C = Alcohol Use Disorders Identification Test-Concise; CI = Confidence interval.

Male				
T_0_	n°	%	AUDIT-C Score Index Mean	95% CI
	321	100	2.330	2.098–2.563
				
Abstainers	93	28.97		
Drinkers	228	71.03	3.281	3.047–3.514
				
Low Risk	148	64.91	2.209	2.076–2.342
Moderate Risk	52	22.81	4.365	4.230–4.501
High Risk	23	10.09	6.696	6.492–6.899
Severe Risk	5	2.19	8.00	8.000–8.000
				
**T_1_**	**n°**	**%**	**AUDIT-C Score Index Mean**	**95% CI**
	321	100	2.835	2.565–3.105
				
Abstainers	66	20.56		
Drinkers	255	79.44	4.141	3.873–4.409
				
Low Risk	112	43.92	2.170	2.011–2.328
Moderate Risk	69	27.06	4.304	4.193–4.416
High Risk	51	20.00	6.392	6.253–6.531
Severe Risk	23	9.02	8.286	8.067–8.455
				
**Female**				
**T_0_**	**n°**	**%**	**AUDIT-C Score Index Mean**	**95% CI**
	319	100	2.389	2.167–2.610
				
Abstainers	80	25.08		
Drinkers	239	74.92	3.181	2.965–3.190
				
Low Risk	150	62.76	2.127	1.991–2.262
Moderate Risk	62	25.94	4.403	4.256–4.501
High Risk	26	10.88	6.500	6.294–6.706
Severe Risk	1	0.42	9	-
				
**T_1_**	**n°**	**%**	**AUDIT-C Score Index Mean**	**95% CI**
	319	100	3.034	2.788–3.281
				
Abstainers	55	17.24		
Drinkers	264	82.76	3.716	3.479–3.953
				
Low Risk	133	50.38	2.113	1.967–2.259
Moderate Risk	75	28.41	4.373	4.261–4.485
High Risk	48	18.18	6.375	6.233–6.517
Severe Risk	8	3.03	8.250	7.863–8.637

## Data Availability

The data are not publicly available due to privacy restrictions.
